# CYP1B1 gene: Implications in glaucoma and cancer

**DOI:** 10.7150/jca.42669

**Published:** 2020-05-19

**Authors:** Arwa Alsubait, Wadha Aldossary, Mamoon Rashid, Amani Algamdi, Bahauddeen M. Alrfaei

**Affiliations:** 1 Biochemisty, King Saud University, Saudi Arabia.; 2 King Abdullah International Medical Research Center (KAIMRC)/ King Saud Bin Abdulaziz University for Health Sciences (KSAU-HS), MNGHA, Saudi Arabia.

**Keywords:** Glaucoma, CYP1B1, cancer, optic nerve, carcinogens, estrogens

## Abstract

Glaucoma is a serious disease that can lead to irreversible loss of vision. Patients with primary congenital glaucoma may have elevated intraocular pressure. Hypertension causes damages to intraocular structures and affects the Schlemm's canal, collector channels, trabecular meshwork, and optic nerve's molecular structures. An important gene that is defective in patients with glaucoma is CYP1B1, a gene associated with optic nerve deterioration. CYP1B1is a key enzyme involved in the metabolism of exogenous and endogenous compounds. Also, it is critical in the detoxification of pre-carcinogens, such as polycyclic aromatic hydrocarbons and estrogen. It catalyzes their conversion into metabolites subsequently eliminated from the body. In malignant tumors, the CYP1B1 promoter is hypomethylated. CYP1B1 overexpression results in the conversion of estrogens to quinone forms, which bind with DNA and create a predisposition for cancer in several organs, such as the brain, breast, and ovary. Increased cytokine interleukin-6 and leptin lead to elevated CYP1B1 activity, which possibly causes cancer. In addition, the expression of aromatic hydrocarbon receptors is increased in tumor tissues, and it elevates oxidative stress and cell growth. TCGA database analysis showed increased survival at bladder and renal carcinoma when CYP1B1 expression is low. Therefore, alteration of CYP1B1 expression may suggest a therapeutic benefit for multiple diseases such as glaucoma and cancer.

## Introduction

Metabolism of endogenous and exogenous compounds depends on cytochrome P450 activity 1,2. The CYP protein was previously called 'cytochrome P450' following the discovery of a colored pigment in the cell that had a 450 nm wavelength spectrum when reduced and bound with carbon monoxide. The name continues to be misused as these proteins are not true cytochromes. They are more appropriately called 'heme-thiolate monooxygenases' 3. CYP enzymes are stimulated by aromatic hydrocarbon receptors (AhRs), and they consist of a large number of proteins. They are involved in the metabolism of xenobiotics and endogenous compounds such as oestradiol 4. They are also responsible for the biotransformation of most foreign substances and drug detoxification 5. CYP expression is affected by many factors, such as sex, age, and genetic polymorphisms. The expression can also be influenced by stimulation from xenobiotics, through modulation by cytokines and hormones, and during disease states 6.

### CYP1B1

Cytochrome P450 (CYPs) are a group of enzymes consist of a large heme prosthetic group catalyzing the oxidation reactions of a wide range of organic substrates. P450 proteins were the first group of enzymes to be classified as a “superfamily” and the total number of enzymes belong to this family is more than 2100 members 7. The cytochrome P450 CYP1 family (CYP1) is responsible for the phase I metabolism of endogenous and exogenous substrates and they are involved in the oxidative metabolism of endogenous materials, such as bile acids, steroid hormones, and lipids. Furthermore, CYP1 proteins metabolize potential carcinogens such as aryl hydrocarbons, aromatic amines, heterocyclic aromatic amines, and heterocyclic amines. CYP1 family has an essential role in procarcinogen activation, catalyzing the metabolism of ~ 66% of potential carcinogens. The biotransformation of procarcinogens causes the creation of mutagenic materials, which form adducts with nucleic bases, responsible for the beginning of carcinogenesis 8.

**Figure 1:**
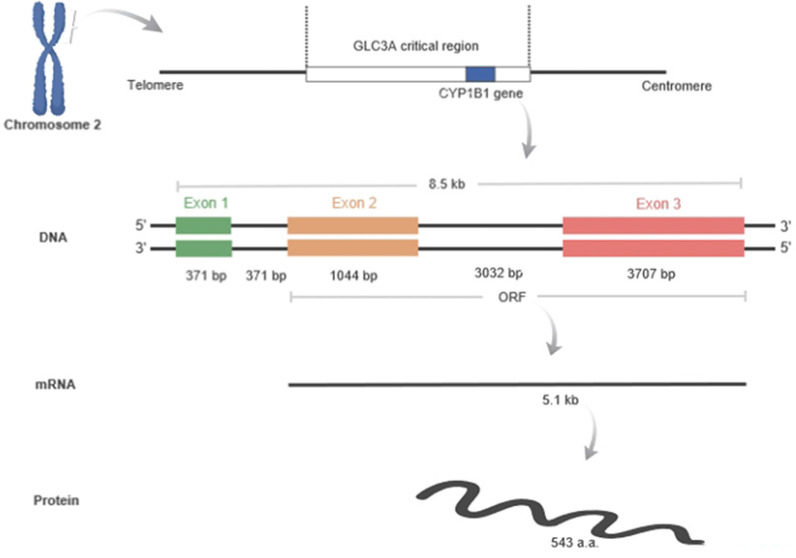
** Human CYP1B1 located at 2p21-22 in the GLC3A critical region.** The length of this gene is 8.5 kilobases, and it contains three exons and two introns: exon1 (371 bp), intron 1 (371 bp), exon 2 (1044 bp), intron 2 (3032 bp), and exon 3 (3707 bp). The open reading frame (ORF) starts from exon 2 and continues to exon 3. The ORF is translated into about 5.1 kb mRNA, which encodes a predicted 543 amino acids. Figure created by Biorender software.

**Figure 2:**
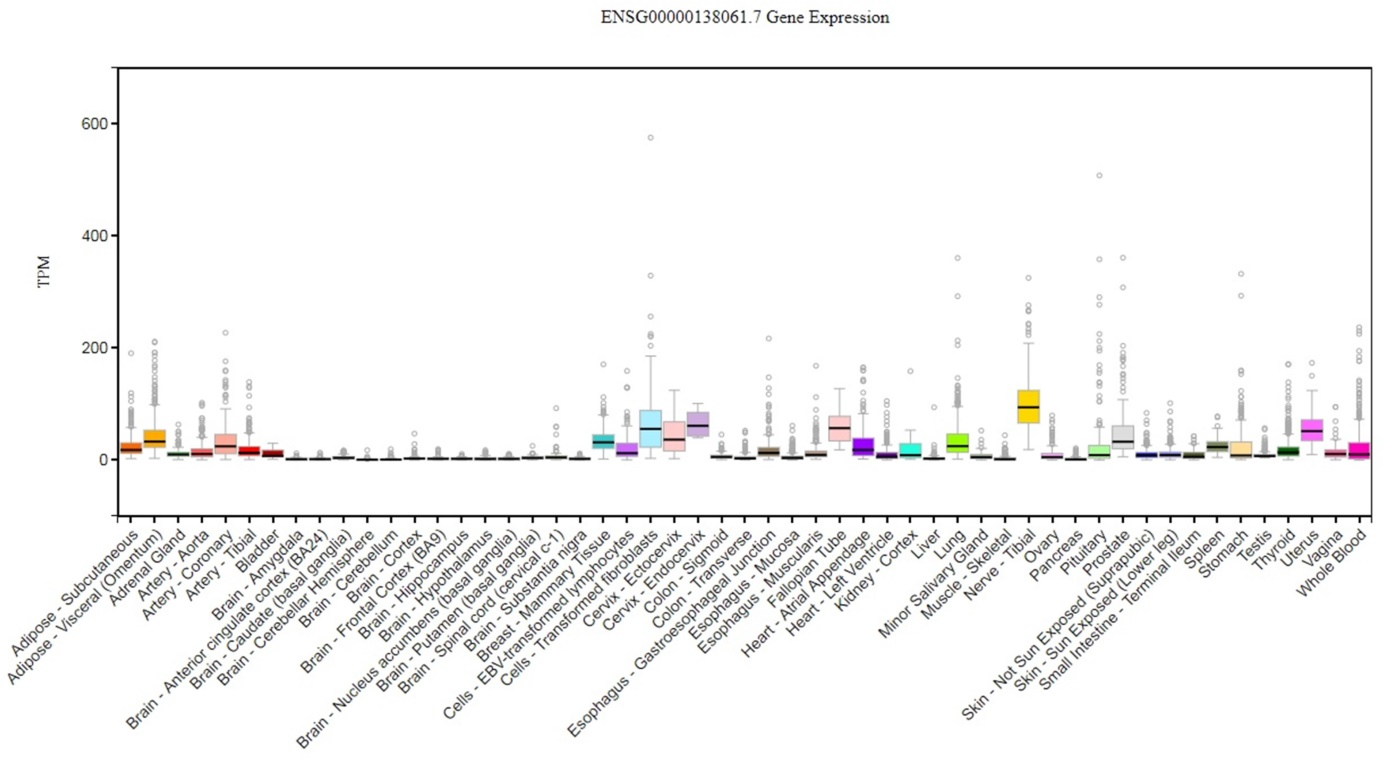
***CYP1B1* gene expression across diverse normal human tissues.** The source of this figure is Genotype-Tissue expression (GTEx) project database, gtexportal.org.

**Figure 3:**
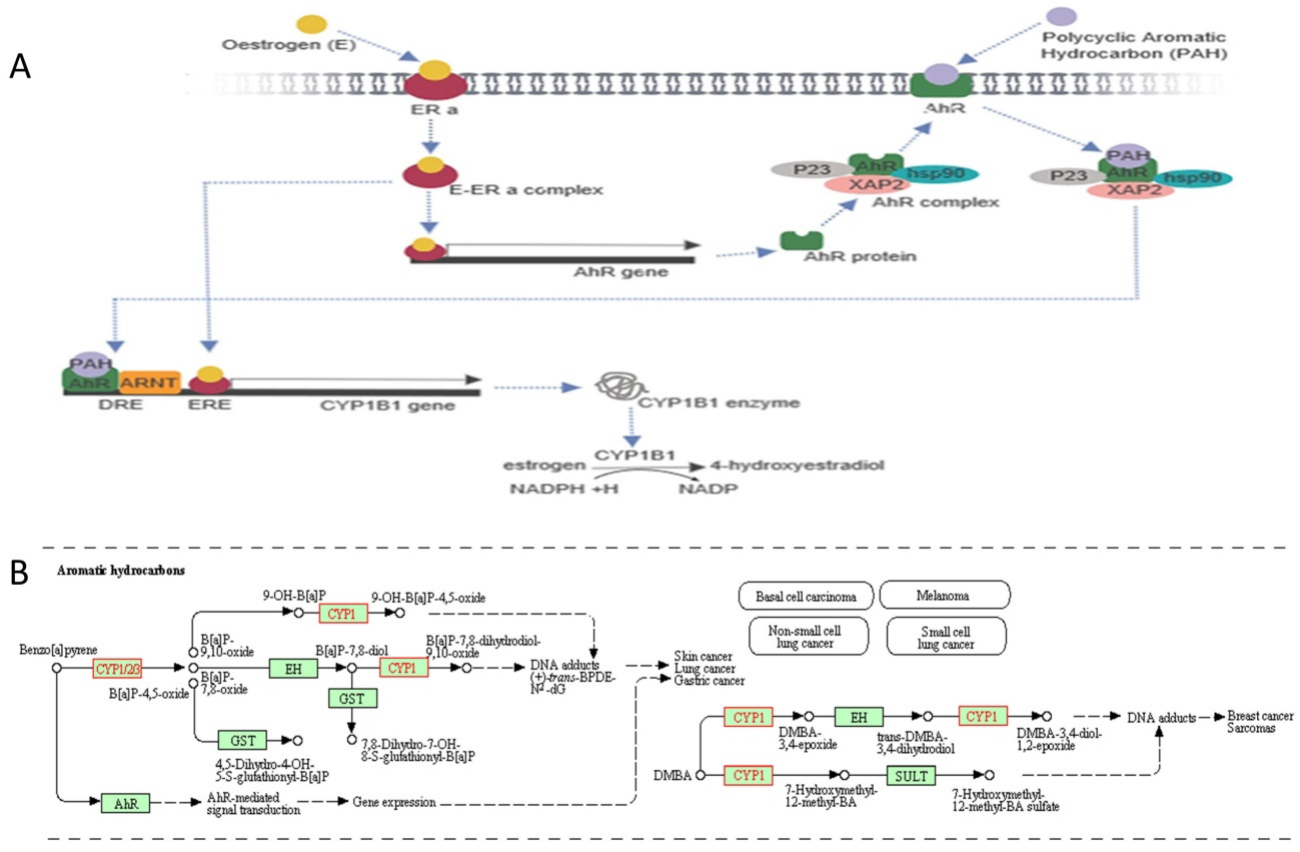
** Aromatic hydrocarbon in conjunction with *CYP1B1* signalling pathway. A,** Oestrogen binds with the oestrogen receptor alpha (ERa) to form an oestrogen-oestrogen receptor alpha complex (E-ERa), which induces an aromatic hydrocarbon receptor (AhR) gene. The AhR protein attaches to heat shock protein-90 (hsp90), XAP2 and P23 to form an AhR complex, then transport into the cell membrane. Also, AhR binds with a polycyclic aromatic hydrocarbon (PAH) ligand to form an AhR complex/PAH complex. The AhR complex dissociates to form a heterodimer, AhR-ligand/ARNT. The heterodimer with oestrogen promotes the CYP1B1 gene. The CYP1B1 enzyme is involved in oestrogen metabolism by converting oestrogen into 4-hydroxyestradiol, which depends on NADPH. The figure created by Biorender software. **B,** CYP1B1 gene involved in carcinogenesis pathway using Aromatic hydrocarbons leading to different types of cancers. The figure obtained from KEGG pathways database.

The cytochrome P450 CYP1 family consists of the CYP1A, CYP1B, CYP1C and CYP1D subfamilies 9-11. Among the human P450 subfamilies, the CYP1B1 gene is the largest known in terms of mRNA size and number of amino acids; it is also the simplest in terms of its structure 9. It is found on chromosome 2p21-22. Its length is approximately 8.5 kilobases (kb) of DNA, consisting of three exons and two introns. The size of its mRNA is 5.1 kb 12,13. It has an open reading frame (ORF) starts at the 5' end of the second exon and encodes a predicted protein of 543 amino acids (**Figure 1**) 12. In humans, CYP1B1 is expressed constitutively in healthy cells including eye, lungs, heart, kidney, prostate, brain, liver, uterus, ovaries and placenta 14-17. It is also expressed in cells of the immune system and estrogen target tissues, such as mammary glands 17,18. CYP1B1 expression in most organs was reported in the GTEx database as of October 21^st^, 2019 using GTEx Analysis version 7 (**Figure 2**). Tissues are known to accumulate melatonin or 6-hydroxymelatonin also has high expression levels of the CYP1B1 protein 19. CYP1B1 is one of the most important enzymes involved in the metabolic activation of pre-carcinogens 1, such as polycyclic aromatic hydrocarbons 20, oxides, amines, and estrogens (**Figure 3A**) 21. Moreover, CYP1B1 polymorphisms have been incriminated as risk factors in different types of cancers and glaucoma 22. Here, we focus on the correlation between CYP1B1 and the development of glaucoma and cancer.

### CYP1B1 in Glaucoma

Glaucoma is a heterogeneous disease that represents the second primary cause of irreversible loss of eyesight worldwide 23, 24. Globally, ~64.3 million individuals are diagnosed with glaucoma. This prevalence is anticipated to increase to 76 million in 2020 25 and 111.8 million in 2040 26. Glaucoma is a nonuniform group of neurodegenerative ocular disorders 27 characterized by several clinical features that include visual field defects, retinal ganglion cell death, and progressive degeneration of the optic nerve 23. The primary source of the progressive degeneration of the optic nerve is the occurrence of a lesion in the axons of the retinal ganglion cells 28. International Classification (CGRN/WGA) of Childhood Glaucoma divided glaucoma into two types: Primary childhood glaucoma and Secondary childhood glaucoma. The primary childhood glaucoma is classified into Primary congenital glaucoma (PCG; OMIM 231300) and Juvenile open-angle glaucoma (JOAG; OMIM 137750). The Secondary childhood glaucoma is classified into four categories: glaucoma associated with nonacquired ocular anomalies, glaucoma associated with nonacquired systemic anomalies, glaucoma associated with the acquired condition, and glaucoma following cataract surgery 29. In children, the most common form is PCG, with a prevalence rate of 1:10,000 to 18,000 live births worldwide 30,31. The frequency of CYP1B1 mutations in people with PCG depends on the population being considered. For example, in Saudi Arabian and Slovakian Roma residents, CYP1B1mutations were detected nearly in all cases of PCG 32. Additionally, the prevalence of mutations in Brazil is 50%, in Indonesia it is 30%, and in Japan, it is 20%. Therefore, variability in prevalence indicates that other main genetic factors for PCG have not been identified yet. PCG is usually inherited as an autosomal recessive disease with incomplete penetrance 33. This form is considered a rare form of glaucoma. It is characterised by isolated angle anomalies, possibly involve mild congenital iris anomalies, ocular hypertension, buphthalmos (enlargement of the eye), oedema, corneal opacification (scarring or clouding of the cornea), descemet's membrane herniation, lower density of the anterior sclera, iris atrophy and increased depth of the anterior chamber. Patients with PCG manifest variable symptoms include photophobia (intolerance to visual observation of light), blepharospasm (involuntary closing of the eyelids), and increased lacrimation (tear discharge) 33. Optic neuropathy is not the sole contributor to PCG; glial cells are also considered major players in disease pathogenicity 34. The development of ocular structures also involves the CYP1B1 enzyme 12, as confirmed by the detection of the CYP1B1 protein in several ocular cells of normal human fetal and adult eyes via immunolocalization assays. The intensity of CYP1B1 expression in fetal compared to adult human eyes indicates much more intense CYP1B1 immunolabeling. Anti-CYP1B1 immunoreactivity was evident in the primitive ciliary epithelium, non-pigmented ciliary epithelium, corneal epithelium, and keratocytes but was absent in the trabecular meshwork in all samples 16. Notably, CYP1B1 was expressed in the human fetal nasal mucosa 35.

Although the diagnosis of PCG depends on clinical findings, the genetic tests could confirm the diagnosis. Several efforts have been initiated to understand the underlying mechanisms of the disease by using genome-wide association studies (GWAS) and genome sequencing technologies. Multiple PCG loci have been mapped; however, only *CYP1B1* has been identified to date as the leading cause of PCG. Interestingly, more than 150 mutations in *CYP1B1*, including missense, nonsense, regulatory, insertions, or deletions, have been associated with PCG 33. In glaucoma, most scientists focus on the optic nerve. However, optic nerve supporting cells and their environments are not trivial. In glaucoma, characterized CYP1B1 mutations have not been properly investigated in astrocytes, microglia, oligodendrocytes, or other neural stromal cells. In PCG, patients usually have a global mutation that increases disease severity at various levels and in a variety of cells. Therefore, scientific awareness should be practiced in areas not covered entirely in the scientific literature, and hopefully, research will be geared towards them in the future.

### CYP1B1 in Cancer

CYP1B1 belongs to a group of enzymes known as Cytochrome P450s 9. Interestingly, CYP1B1 is one of the main enzymes that are required in the hydroxylation of estrogens and the activation of some carcinogens. CYP1B1 mediates the metabolic activity of many procarcinogens. Polycyclic aromatic hydrocarbons (PAHs) are an example of a procarcinogen in pulmonary cells that can be converted to carcinogenic metabolites capable of producing DNA adducts (**Figure 3B**). For instance, quinones react with the N-7 of guanine and N-3 of adenine in DNA. This reaction generates DNA adducts that are capable of producing DNA mismatches during the DNA replication process, as well as modifying promoter methylation or promoter binding, leading to an inheritable DNA mutation or irregular gene expression, and eventually tumorigenesis. The reactive metabolites of PAHs may also motivate the formation of protein adducts in cells, which may influence the normal activities of these proteins. Additionally, PAH metabolites may also increase the production of reactive oxygen species (ROS), which can affect DNA, lipids, or proteins and commence carcinogenesis 36.

Furthermore, up-regulation of CYP1B1 is an indication of cancer, since it is highly selectively expressed in cancerous rather than healthy tissues 37. High CYP1B1 expression is found in multiple malignant tumors, including those of the brain, breast, colon, ovarian, and prostate cancers 9,38. Immunohistochemical investigations have confirmed CYP1B1 overexpression in a majority of ovarian cancers. Most primary ovarian cancers (92%) show CYP1B1 immunoreactivity, present in the cytoplasm of the tumor cells. It is also detected in a majority (94%) of metastatic ovarian cancers. Some groups consider the CYP1B1 protein to represent an anticancer drug 39. In a study done by Kwon and Chun on different cell lines, including MCF-7, MDA-MB-231, and HeLa cells, they reported that CYP1B1 increased invasion and inhibited cancer cell apoptosis 40. Other studies have reported the effects of CYP1B1 knockout in vivo. When carcinogenic materials were delivered into mice with CYP1B1 knockout, decreased tumor growth, and increased DNA adduct formation was observed. These reports strongly suggest that CYP1B1 is a promising cancer biomarker and a potential target for the development of anticancer drugs 41. However, a comprehensive molecular mechanism describing how CYP1B1 contributed to oncogenesis remains unknown 40.

### Molecular mechanism of CYP1B1

Wnt/β-catenin signaling may be associated with epithelial-mesenchymal transition (EMT) because they both need β-catenin 42. To understand CYP1B1 molecular mechanisms, multiple studies have measured key oncogenic proteins including β-catenin, c-Myc, and ZEB-2 after CYP1B1 modulation. Jung Kwon et al. explored the role of CYP1B1 in cancer development and progression, trying to find out the leading molecular players that drive CYP1B1-mediated oncogenesis. β-catenin plays a vital role in mediating the Wnt/β-catenin signaling pathway. Upon the binding of the Wnt ligand to the receptor, β-catenin escapes the proteasomal degradation and trans locates into the nucleus, where it unites with its target genes and activates various signaling pathways involved in carcinogenesis. Furthermore, Jung Kwon and colleagues measured different markers of tumor progression, including cell proliferation, invasion, and cell migration after CYP1B1 modulation, induction, or inhibition. They observed multiple target proteins, including CTNNB1, ZEB2, SNAIL1, and TWIST1, that are correlated with EMT and Wnt/β-catenin signaling were upregulated. Their findings indicate that CYP1B1 induces EMT and activates Wnt/β-catenin signaling via upregulating Sp1, a transcription factor that participates in cell proliferation and metastasis. This cascade of cellular actions inhibited E-cadherin expression and increased Wnt/β-catenin signaling upon the upregulation and nuclear translocation of β-catenin. Therefore, this was the first published study that reported the molecular mechanisms of how CYP1B mediates tumorigenesis 40. A more detailed understanding of the underlining mechanisms of CYP1B1-mediated carcinogenesis may help in the development of new methods for cancer treatment.

Sex hormone metabolism has defected in mammary gland tumorigenesis, and variable metabolites may have different effects on mammary epithelial cells. 4-hydroxy estradiol (4-OHE2), an oxidized metabolite of estrogen, is significantly expressed in human breast cancer cells. This catechol estrogen has been proposed as an inducer of hormonal carcinogenesis, such as breast cancer. Furthermore, when transgenic mice with increased expression of CYP1B1, an essential enzyme of 4-hydroxy metabolites 1, were stimulated with estrogen, cancer cells were found in their mammary glands 43.

Hypomethylation in the promoter region of CYP1B1 regulates the overexpression of this gene, as determined in clinical prostate tissues and prostate cancer cell lines using methylation-specific PCR and bisulfite-modified DNA sequencing 44. Another study investigated the function of miRNA in drug-metabolizing enzymes provided another reason for elevated CYP1B1 levels. The results of luciferase assays suggested that miR-27b regulates CYP1B1 at the post-transcriptional stage. A decreased level of miR-27b was associated with elevated levels of CYP1B1 protein in cancerous tissues 45. Recently, another miRNA, miR-187-5p, was also shown to have decreased expression in non-small lung cancer. Interestingly, CYP1B1 is a direct target gene for miR-187-5p, which is expressed during cancer cell growth 46.

Moreover, elevated levels of the pro-inflammatory cytokine interleukin-6 also have effects on CYP1B1 expression in HCT116 and SW480 colorectal cancer cell lines. This cytokine can manipulate CYP1B1 expression and can induce the activation of dietary carcinogens and DNA damage that can trigger colorectal cancer progression 47. Additionally, the breast cancer microenvironment can be guided by leptin, which directly stimulates estrogen metabolism genes, including CYP1B1, that are involved in the oxidative metabolism (4-hydroxylation) of estrogen. Oxidative metabolism of estrogen leads to DNA adduct formation and cancer mutations 48.

Cigarette smoke carcinogens such as 4-(methylnitrosamino)-1-(3-pyridyl)-1-butanone (NNK) and polycyclic aromatic hydrocarbons (PAH) are known as significant causes of lung cancer, which kills on average 3000 people per day worldwide 49. NNK and PAH upregulate CYP1B1 enzyme activity upon binding to AhR (**Figure 3B**). The AhR overexpression upregulated the expression of CYP1B1 in the early stage of lung adenocarcinoma, which could increase intracellular oxidative stress and promote cell growth. Therefore, the disruption of AhR expression could prevent the early development of lung adenocarcinomas 50.

Flavonoids are a group of common substances with variable phenolic structures. These compounds are found in natural products such as fruits, vegetables, grains, blossoms, tea, and wine. These particular items are well known for their beneficial impacts on wellbeing, and tremendous efforts are being made to isolate Flavonoids from these natural products. Because of Flavonoids anti-oxidative, anti-inflammatory, and anti-carcinogenic properties and due to their ability to balance main cellular enzyme functions, they are considered as an essential component in multiple nutraceuticals, pharmaceutical, medicinal, and cosmetic uses 51. Flavonoids have a tight, rigid structure that enables them to fit precisely within the active site of the CYP1B1 enzyme 21.

Interestingly, dietary flavonoids can be metabolized by CYP1B1 to products that can inhibit cancer proliferation 52. Furthermore, many studies recommended the use of flavonoids to treat several types of cancer, such as melanoma, hepatocellular carcinoma, and thyroid cancer. The administration of flavonoid in animal models of cancer has enhanced the tolerance to cancer treatment 51.

Natural flavonoids are the primary source of CYP1B1 inhibitors. Methoxy types of flavones and flavonols were appeared to prevent CYP1B1 activity, such as chrysoeriol and isorhamnetin specifically. Various flavonoids including quercetin, rutin, apigenin, and amentoflavone, show an inhibitory effect of CYP1B1. Some CYP1B1 inhibitors, such as kaempferol and isorhamnetin, can also antagonize the expression of AHR, which may show synergetic inhibition on the expression of CYP1B1. Interestingly, some anti-cancer drugs used in clinics are competitive inhibitors of CYP1B1, such as flutamide, paclitaxel, mitoxantrone, and docetaxel. CYP1B1 inhibitors can be utilized to understand the CYP1B1 function; thus, it might be considered as anti-cancer agents for the treatment of certain types of cancer 53.

In cancer cells, CYP1B1 is highly expressed. This observation proposes that CYP1B1 enzyme may metabolize variable anticancer drugs. Cisplatin is considered an essential chemotherapeutic agent, but it might also play a role in chemo-resistance. The presence of CYP1B1 in tumor cells decreases the sensitivity of the cells to the cytotoxic effects of Cisplatin. However, the co-administration of potent, cell permeable and water soluble CYP1B1 inhibitors, such as the synthetic pyridylchalcones 7k (DMU2105) and 6j (DMU2139), with Cisplatin can overcome CYP1B1-mediated resistance and re-sensitized cancerous cells once again to the Cisplatin 54. These findings suggest the useful roles of CYP1B1 inhibitors in the treatment of cancer.

### Prognosis and interactions of CYP1B gene

**Figure 4:**
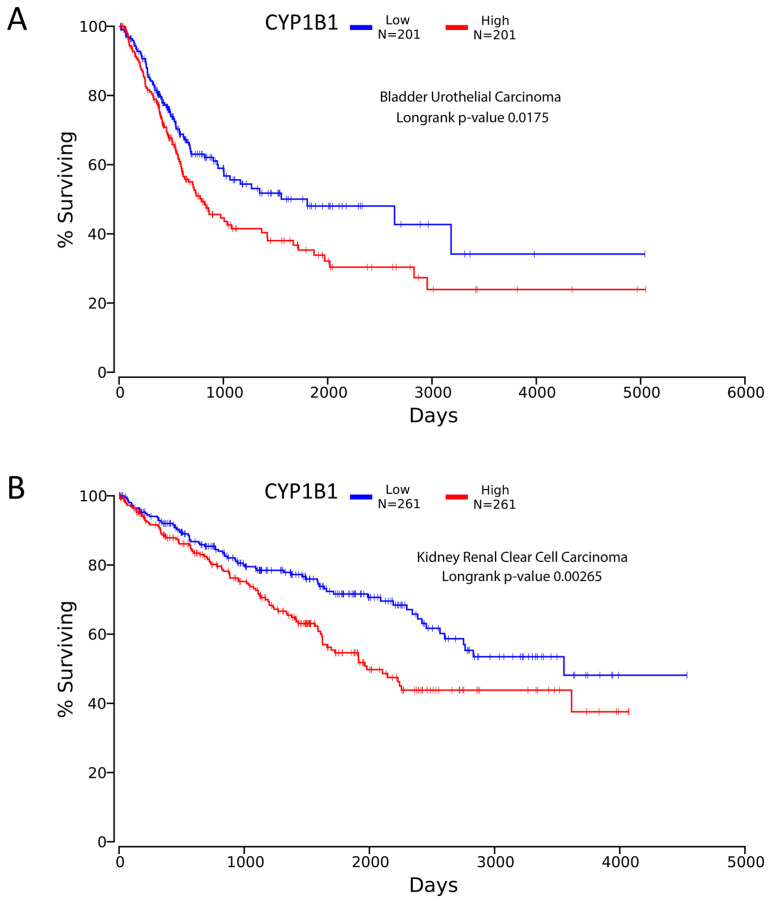
** TCGA survival curves using Kaplan meier analysis. A,** represents bladder urothelial carcinoma showing longer survival for low expression CYP1B1 patients. **B,** represents renal clear cell carcinoma showing extended survival for decreased expression CYP1B1 patients. Figures created from oncolnc.org website with TCGA database incorporated.

Looking at TCGA database, significance was found for CYP1B1 expression relevant to bladder and kidney carcinoma. Low expression of CYP1B1 in cancer corresponded to better survival detected by Kaplan-meier plot for kidney (renal) clear cell carcinoma and bladder urothelial carcinoma with p-value 0.00265 and 0.0175 respectively (**Figure 4**). The data was extracted from over 200 patients suffering from cancer in both diseases 55. This finding opens the door for scientists to investigate possible treatment methods such as CYP inhibitors to achieve better survival in both cancers.

**Figure 5:**
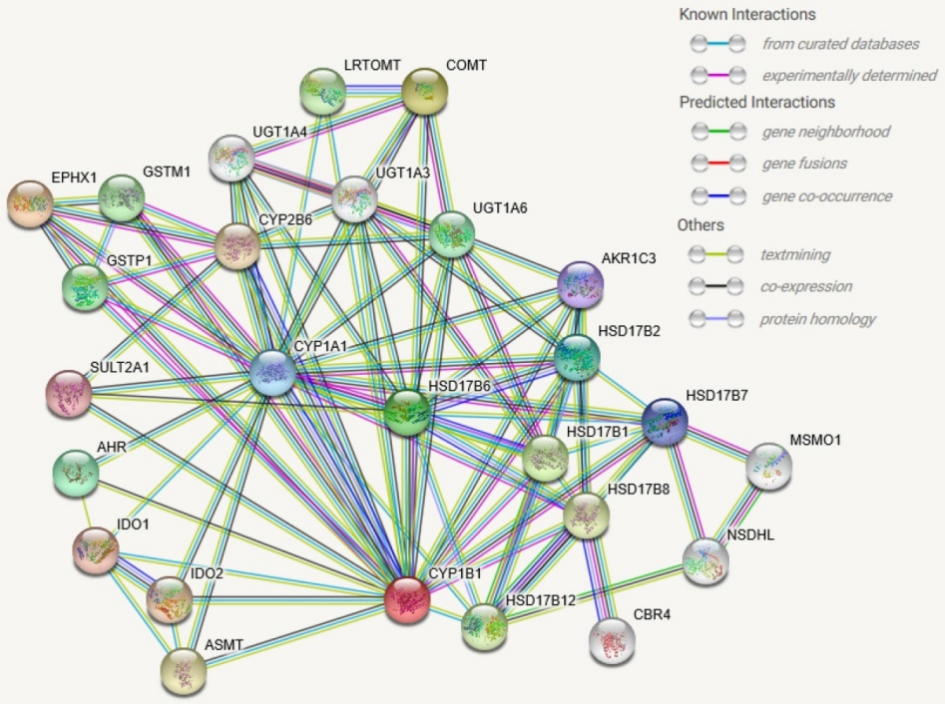
** CYP1B1 protein-protein associations frpm STRING database.** Colored nodes are query proteins and first shell of interactors. White nodes are second shell of interactors. filled nodes (structure inside the node) represent some 3D structure is known or predicted. Interactions include: i) known interactions from curated databases and experimentally determined, ii) predicted interactions by gene neighborhood, gene fusion, and gene co-occurrence, iii) others by text mining, co-expression, and protein homology. All interactions have been segregated by different colors.

Interaction of CYP1B1 genes have been documented in many research papers through frpm STRING database. Level of confidence was assessed based on physical experiment, bioinformatics predictions, co-expressions anticipation and indirect association, See **Figure 5**. These interactions involve biological processes, molecular functions, and/or cellular components. The STRING database has identified 100 publications for CYP1B1 interactions and minimum 22 pathways when scale of confidence was set at high confidence. This information will enhance the scientific investigation toward validating predicted phenomenon or interaction. In addition, **Table 1.** lists functions of molecules that directly interact with CYP1B1.

## Conclusion

High-advanced technologies, including whole-exome sequencing (WES) and whole-genome sequencing (WGS), helped to reveal the molecular mechanisms of how genetic abnormalities such as CYP1B1 mutations lead to glaucoma and cancer. Glaucoma-related abnormalities are severe and can cause irreversible blindness. The CYP1B1 gene is the most common gene mutated in PCG. Although many articles have pointed out the importance of this gene, molecular and physiological information are not completed yet. Due to the lack of research support, many mutations have been characterized, but others have been neglected. Also, some African and Middle-Eastern mutations are not characterized yet.

Furthermore, scientists are investigating either CYP1B1 mutations or CYP1B1 overexpression since they contribute to glaucoma or cancer, respectively. On the other hand, a balanced expression of CYP1B1 in either glaucoma or cancer has not been investigated. Thus, multiple questions remain unanswered, such as: does high expression in the eye cause cancer? Or does a reduction of expression in other organs, such as the liver, create difficulty? We have promising disease candidates for CYP1B1 inhibitor trials which are kidney renal clear cell carcinoma and bladder urothelial carcinoma. Additional information is needed to evaluate different treatment plans in those scenarios. With the development of new genetic engineering techniques, a possible option of treatment is the correction of the CYP1B1 abnormality via gene-editing technologies such as the CRISPR-Cas9 system.

Additionally, genome-wide association studies (GWAS) and other cellular techniques studying gene expression and regulation will undoubtedly change our understanding of how CYP1B1 mutations contribute to the pathogenesis of glaucoma and cancer. In order to achieve this, in the future, it is essential to have full access to glaucoma and cancer patient's samples. Also, it is vital to have an available platform occupied with all instrumental technologies needed to perform different research studies such as functional genomics, gene expression studies, pathway analysis, and epigenetic studies. Therefore, the ultimate knowledge we will get out from these studies will expand our understanding of the different cellular and molecular mechanisms of glaucoma and cancer, which in turn will help in the development of new therapeutic options for glaucoma and cancer patients.

## Figures and Tables

**Table 1:** List of direct interactive items with CYP1B1

No.	Abbreviation	Names of Direct interactive items with CYP1B1 in Figure 5
1	EPHX1	Epoxide hydrolase 1; Biotransformation enzyme that catalyzes the hydrolysis of arene and aliphatic epoxides to less reactive and more water soluble dihydrodiols by the trans addition of water (By similarity). May play a role in the metabolism of endogenous lipids such as epoxide-containing fatty acids.
2	COMT	Catechol O-methyltransferase; Catalyzes the O-methylation, and thereby the inactivation, of catecholamine neurotransmitters and catechol hormones. Also shortens the biological half-lives of certain neuroactive drugs, like L-DOPA, alpha-methyl DOPA and isoproterenol; Seven-beta-strand methyltransferase motif containing.
3	HSD17B1	Estradiol 17-beta-dehydrogenase 1; Favors the reduction of estrogens and androgens. Also has 20-alpha-HSD activity. Uses preferentially NADH; Short chain dehydrogenase/reductase superfamily.
4	HSD17B6	17-beta-hydroxysteroid dehydrogenase type 6; NAD-dependent oxidoreductase with broad substrate specificity that shows both oxidative and reductive activity (in vitro). Has 17-beta-hydroxysteroid dehydrogenase activity towards various steroids (in vitro). Converts 5-alpha-androstan-3- alpha,17-beta-diol to androsterone and estradiol to estrone (in vitro). Has 3-alpha-hydroxysteroid dehydrogenase activity towards androsterone (in vitro). Has retinol dehydrogenase activity towards all-trans-retinol (in vitro).
5	AHR	Aryl hydrocarbon receptor; Ligand-activated transcriptional activator. Binds to the XRE promoter region of genes it activates. Activates the expression of multiple phase I and II xenobiotic chemical metabolizing enzyme genes (such as the CYP1A1 gene). Mediates biochemical and toxic effects of halogenated aromatic hydrocarbons. Involved in cell-cycle regulation. Likely to play an important role in the development and maturation of many tissues.
6	HSD17B2	Estradiol 17-beta-dehydrogenase 2; Capable of catalyzing the interconversion of testosterone and androstenedione, as well as estradiol and estrone. Also has 20-alpha-HSD activity. Uses NADH while EDH17B3 uses NADPH; Short chain dehydrogenase/reductase superfamily.
7	CYP1A1	Cytochrome P450 1A1; Cytochromes P450 are a group of heme-thiolate monooxygenases. In liver microsomes, this enzyme is involved in an NADPH-dependent electron transport pathway. It oxidizes a variety of structurally unrelated compounds, including steroids, fatty acids, and xenobiotics.
8	HSD17B7	3-keto-steroid reductase; Responsible for the reduction of the keto group on the C-3 of sterols; Short chain dehydrogenase/reductase superfamily.
9	AKR1C3	Aldo-keto reductase family 1 member C3; Catalyzes the conversion of aldehydes and ketones to alcohols. Catalyzes the reduction of prostaglandin (PG) D2, PGH2 and phenanthrenequinone (PQ) and the oxidation of 9-alpha,11-beta- PGF2 to PGD2. Functions as a bi-directional 3-alpha-, 17-beta- and 20-alpha HSD. Can interconvert active androgens, estrogens and progestins with their cognate inactive metabolites.
10	SULT2A1	Bile salt sulfotransferase; Sulfotransferase that utilizes 3'-phospho-5'-adenylyl sulfate (PAPS) as sulfonate donor to catalyze the sulfonation of steroids and bile acids in the liver and adrenal glands; Sulfotransferases, cytosolic.
11	IDO1	Indoleamine 2,3-dioxygenase 1; Catalyzes the first and rate limiting step of the catabolism of the essential amino acid tryptophan along the kynurenine pathway. Involved in the peripheral immune tolerance, contributing to maintain homeostasis by preventing autoimmunity or immunopathology that would result from uncontrolled and overreacting immune responses. Tryptophan shortage inhibits T lymphocytes division.
12	IDO2	Indoleamine 2,3-dioxygenase 2; Catalyzes the first and rate limiting step of the catabolism of the essential amino acid tryptophan along the kynurenine pathway. Involved in immune regulation. May not play a significant role in tryptophan-related tumoral resistance.
13	CYP2B6	Cytochrome P450 2B6; Cytochromes P450 are a group of heme-thiolate monooxygenases. In liver microsomes, this enzyme is involved in an NADPH-dependent electron transport pathway. It oxidizes a variety of structurally unrelated compounds, including steroids, fatty acids, and xenobiotics. Acts as a 1,4-cineole 2-exo-monooxygenase
14	ASMT	Acetylserotonin O-methyltransferase; Isoform 1 catalyzes the transfer of a methyl group onto N-acetylserotonin, producing melatonin (N-acetyl-5- methoxytryptamine). Isoform 2 and isoform 3 lack enzyme activity; Pseudoautosomal region 1.
15	HSD17B8	Estradiol 17-beta-dehydrogenase 8; NAD-dependent 17-beta-hydroxysteroid dehydrogenase with highest activity towards estradiol. Has very low activity towards testosterone. The heterotetramer with CBR4 has NADH-dependent 3-ketoacyl-acyl carrier protein reductase activity, and thereby plays a role in mitochondrial fatty acid biosynthesis.
16	HSD17B12	Very-long-chain 3-oxoacyl-CoA reductase; Catalyzes the second of the four reactions of the long- chain fatty acids elongation cycle. This endoplasmic reticulum- bound enzymatic process, allows the addition of two carbons to the chain of long- and very long-chain fatty acids/VLCFAs per cycle.
17	LRTOMT	Transmembrane O-methyltransferase; Catalyzes the O-methylation, and thereby the inactivation, of catecholamine neurotransmitters and catechol hormones (By similarity). Required for auditory function. Component of the cochlear hair cell's mechanotransduction (MET) machinery.
18	GSTM1	Glutathione S-transferase Mu 1; Conjugation of reduced glutathione to a wide number of exogenous and endogenous hydrophobic electrophiles; Soluble glutathione S-transferases
19	GSTP1	Glutathione S-transferase P; Conjugation of reduced glutathione to a wide number of exogenous and endogenous hydrophobic electrophiles. Regulates negatively CDK5 activity via p25/p35 translocation to prevent neurodegeneration; Soluble glutathione S-transferases
20	UGT1A6	UDP-glucuronosyltransferase 1-6; UDPGT is of major importance in the conjugation and subsequent elimination of potentially toxic xenobiotics and endogenous compounds. This isoform has specificity for phenols.
